# Removal of Sodium Dodecylbenzenesulfonate by Macroporous Adsorbent Resins

**DOI:** 10.3390/ma11081324

**Published:** 2018-07-31

**Authors:** Jongho Kim, Daewon Kim, Young Jin Gwon, Kune-Woo Lee, Taek Seung Lee

**Affiliations:** 1 Organic and Optoelectronic Materials Laboratory, Department of Organic Materials Engineering, Chungnam National University, Daejeon 34134, Korea; jh-kim@cnu.ac.kr (J.K.); daewonkim@cnu.ac.kr (D.K.); youngjin@cnu.ac.kr (Y.J.G.); 2 Decontamination and Decommissioning Research Division, Korea Atomic Energy Research Institute, Daejeon 34057, Korea; nkwlee@kaeri.re.kr

**Keywords:** macroporous polymer resins, sodium dodecylbenzenesulfonate, Cs ions, radioactive liquid wastes

## Abstract

Among the surfactants used for removal of radioactive nuclides generated from nuclear decontamination, sodium dodecylbenzenesulfonate (SDBS) is frequently used. A potential environmental problem of incomplete elimination of SDBS from radioactive liquid wastes (RLWs), which contain a high concentration of SDBS and radioactive nuclides, still remains. Removal of SDBS was evaluated by adsorption using commercially available, macroporous polymer-based adsorbents, HP20 and HP2MGL, which are styrene (St)- and methyl methacrylate (MMA)-based crosslinked resin beads, respectively. The effect of the macroporosity and chemical structure of the macroporous adsorbent resins (MARs) on the adsorption behavior was investigated. HP20 did not have any functionality for adsorbing SDBS, but it showed effective adsorption toward SDBS (less than 20 min to reach 90% adsorption), because of the hydrophobic interaction between the phenyl groups in the St unit in HP20 and in the SDBS. The removal of SDBS from a mixed solution of SDBS and Cs ions was investigated to elucidate the adsorption process in an imitation of the sort of RLWs. This investigation suggests that MARs can potentially be used for the removal of SDBS not only from a SDBS solution but also from a mixed solution of SDBS and Cs ions.

## 1. Introduction

Huge amounts of radioactive liquid wastes (RLWs) are generated during the operation of nuclear power plants. In addition, such RLWs include enormous amounts of various kinds of surfactants used to remove radioactive nuclides during decontamination processes [1,2,3]. One of the general methods for treating RLWs is the membrane separation method. Although membrane separation has advantages such as low cost, good separation capacity, and reusability, membrane fouling (the adsorption of organic materials, including surfactants) still limits the versatility of this process [4,5,6]. Thus, more convenient separation methods to treat RLWs are urgently required to improve the efficiency of purification processes for RLWs.

Among the surfactants used for removal of radioactive nuclides generated from decontamination and decommissioning, SDBS is one of the anionic surfactants that are frequently used. Although most of the surfactants containing SDBS in domestic waste water (concentration range: 1–10 mg/L) can be completely removed in liquid-waste treatment facilities, a potential environmental problem of incomplete elimination of SDBS from RLWs, which contain a high concentration of SDBS and radioactive nuclides, still remains [7,8]. Because of the toxicity of SDBS, water contaminated with the surfactant including drinking water [9] or RLWs should be purified to discharge clean water to the environment. Although a number of studies have reported the removal of SDBS mixed with metal ions, the removal of SDBS mixed with radioactive nuclides has rarely been studied. Various inorganic materials with high porosity, such as activated carbon [10,11], zeolite [12], magnetic materials [13], and carbon nanotubes [14] have been used for adsorbing SDBS from aqueous solutions. Porous polymeric resins have many advantages over such porous inorganic materials, for example, low cost, high adsorption capability, high porosity, large surface areas, and easy synthesis and surface modification [15]. Such advantages of MARs have resulted in various applications, including in the biomedical field [16] and for separation [17,18], catalysis [19,20], and adsorption [21,22]. Despite the diverse applications of MARs, the removal of surfactants using the MARs has rarely been reported.

In this work, two kinds of MARs that did not contain an ionic functional group (sulfonic acid or ammonium salt) were used to adsorb SDBS in aqueous solution and in an imitation of RLWs containing SDBS at high concentration. The MARs were commercial products with the trade names Dianion HP20 and Dianion HP2MGL, suggesting that they are cost effective. They have different chemical structures, crosslinked polystyrene (PSt) for HP20 and crosslinked poly(methyl methacrylate) (PMMA) for HP2MGL. The adsorption behavior of SDBS on the MARs was investigated in terms of measuring the effects of adsorption time, initial SDBS concentration, and amount of adsorbents used. The adsorption of SDBS mixed with Cs ions, simulating water-based radioactive waste, was investigated using the MARs as a test of this possible practical application.

## 2. Experimental

### 2.1. Materials

Two types of synthetic MARs (Dianion HP20 and Dianion HP2MGL) were purchased from Sigma-Aldrich (Ronkonkoma, NY, USA). SDBS and CsCl were purchased from Sigma-Aldrich and used as received without further purification. NaOH and HCl were purchased from Samchun (Seoul, Korea).

### 2.2. Instrumentation

The Brunauer–Emmett–Teller (BET) surface area and porosity of the adsorbent resins were investigated by nitrogen adsorption at 77 K using a TriStar II3020 V2.00 instrument (Micrometrics Instrument Corp., Norcross, GA, USA). A high-resolution field-emission scanning electron microscope (FE-SEM, JSM-700F, Jeol, Tokyo, Japan) was used to obtain SEM images of the MARs. Fourier transform infrared (FT-IR) spectra were obtained on a Bruker Tensor 27 FT-IR spectrometer (Bruker Optics Inc., Billerica, MA, USA) using KBr pellets in the wavenumber range of 500–4000 cm^−1^. Inductively coupled plasma mass spectroscopy (ICP-MS) was performed using an ELAN DRC II (PerkinElmer SCIEX Waltham, MA, USA) instrument to determine the concentration of Cs ions before and after treatment with the MARs.

### 2.3. Determination of SDBS Concentration

E THSDBS concentration was determined using an ultraviolet-visible (UV-vis) spectrometer at 224 nm (PerkinElmer Lambda 35 spectrometer). UV-vis spectroscopy was carried out in a quartz cell (3.5 mL) with a path length of 10 mm. A calibration curve was established using SDBS concentrations in the range of 3–35 μg/mL.

### 2.4. Batch Adsorption

The removal efficiency of the MARs was investigated under various conditions of adsorption time, initial SDBS concentration (0.1–5 mg/mL for SDBS solutions or 0.1–10 mg/mL for mixed solutions of SDBS and Cs ions), and adsorbent resin concentration (1–50 mg/mL). After a predetermined adsorption time, an aliquot of SDBS solution (0.5 mL) was removed from the solution and diluted with water (2.5 mL). The SDBS concentration in the diluted SDBS solution was determined with UV–vis spectrometer. The amount of SDBS adsorbed on the adsorbent resin, *q* (mg/g), was calculated according to the following equation:

(1) $$q=\left(C_{0}-C\right)\times \frac{\nu }{m}$$

where, *C*_0_ (mg/mL) is the initial SDBS concentration, *C* (mg/mL) is the SDBS concentration after predetermined adsorption time. *v* (mL) is the solution volume and *m* (mg) is the amount of the macroporous adsorbent resin used for SDBS removal.

### 2.5. Desorption Experiment

Desorption experiment was carried using MARs after SDBS adsorption (0.1 mg/mL) for 7 h in aqueous solution. The SDBS loaded MARs were transferred to ultrapure water (10 mL) of various pHs (2, 6, and 10) and then the solution was stirred for 2 h. After the desorption process, an aliquot of solution (0.5 mL) was removed from the solution and diluted with water (2.5 mL). The SDBS concentration in the diluted SDBS solution was determined with UV–vis spectrometer.

## 3. Results

### 3.1. Characterization of the MARs

**Scheme 1:**
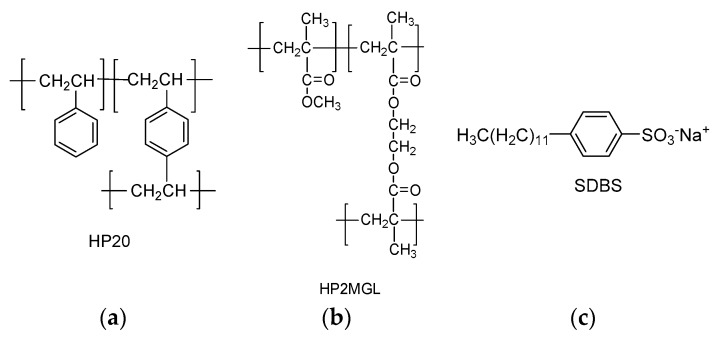
Chemical structures of (**a**) HP20; (**b**) HP2MGL; and (**c**) SDBS.

**Figure 1:**
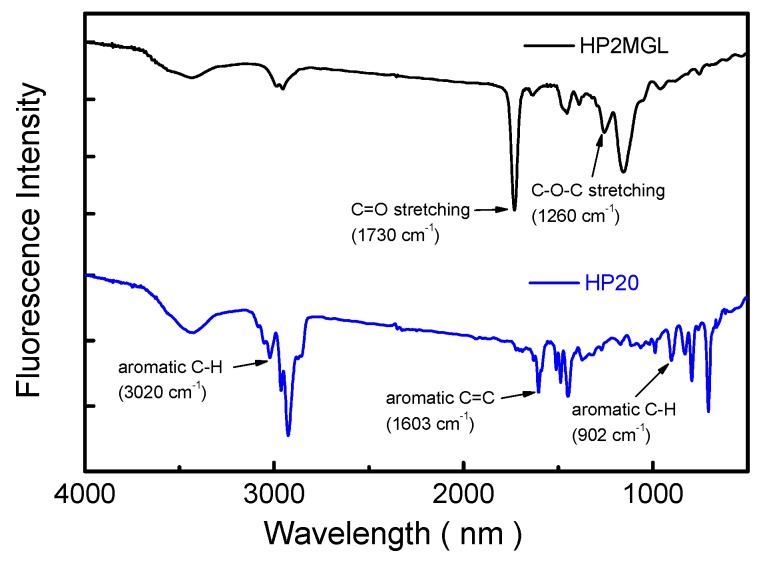
FT-IR spectra of HP20 and HP2MGL.

**Figure 2:**
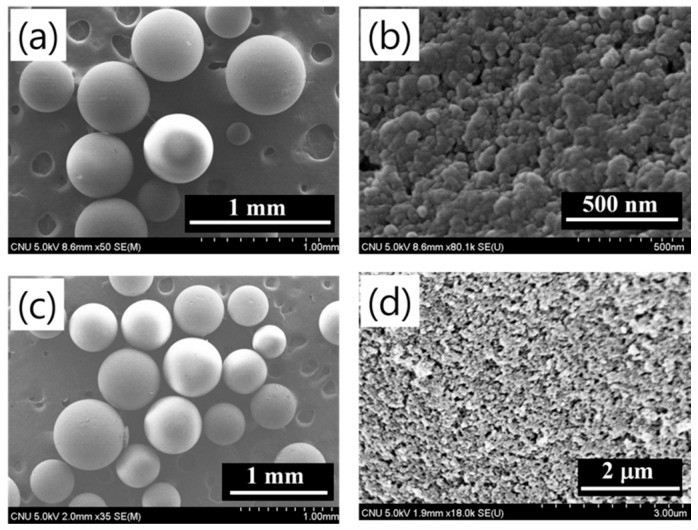
SEM images of (**a**,**b**) HP20 and (**c**,**d**) HP2MGL.

HP20 is composed of a copolymer of St and divinylbenzene (DVB), and HP2MGL of MMA and ethylene glycol dimethacrylate (EGDMA), in which DVB and EGDMA act as crosslinking agents (Scheme 1). To verify the chemical structure, both crosslinked resins were characterized by FT-IR spectroscopy (Figure 1). For HP20, the characteristic band of the aromatic C=C stretching vibration was shown at 1603 cm^−1^. The characteristic band located at 902 cm^−1^ was ascribed to the aromatic C–H deformation vibration. The characteristic band of the C–H stretching vibration of HP2MLG appeared at 3020 cm^−1^ and the characteristic bands located at 1730 and 1260 cm^−1^ were ascribed to the C=O and C–O–C stretching vibrations, respectively. These features clearly indicated the chemical structures of both MARs, which are based on St and MMA. The surface morphology of the resins was investigated with the SEM, and both resins had macroporous structures (Figure 2). The macroporosity was evaluated by the BET method, and both MARs showed a similar surface area, pore volume, and pore radius (Table 1). The MARs used in this study had different chemical structures but similar morphologies, implying that the chemical structure was a major factor in the adsorption of SDBS, and that the physisorption was dominant in the adsorption process.

### 3.2. Effect of Adsorption Time

**Figure 3:**
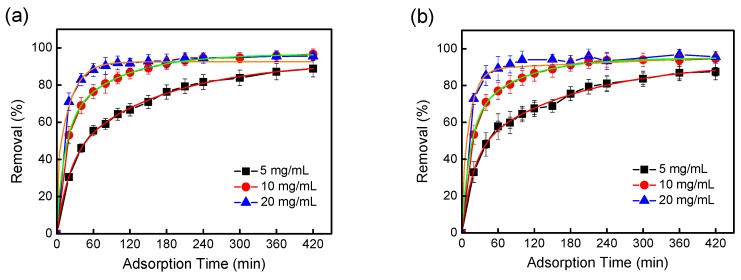
Changes in SDBS adsorption efficiency of (**a**) HP20 and (**b**) HP2MGL with adsorption time at various amounts of adsorbents. [SDBS] = 0.1 mg/mL.

The variation in the efficiency of SDBS removal by the MARs according to adsorption time is shown in Figure 3. The efficiency of SDBS removal using HP20 and HP2MGL increased rapidly and became saturated after around 40 min adsorption time. When both adsorbents (20 mg/mL) were used, 82% SDBS removal was achieved before 40 min, followed by 90% after 90 min at an SDBS concentration of 0.1 mg/mL (100 ppm). The initial adsorption occurred in the macropores of the MARs in the early stages of adsorption, and the rate of adsorption decreased to the equilibrium, indicating that the macropores were easily filled with SDBS.

### 3.3. Effect of the Amount of Adsorbents

**Figure 4:**
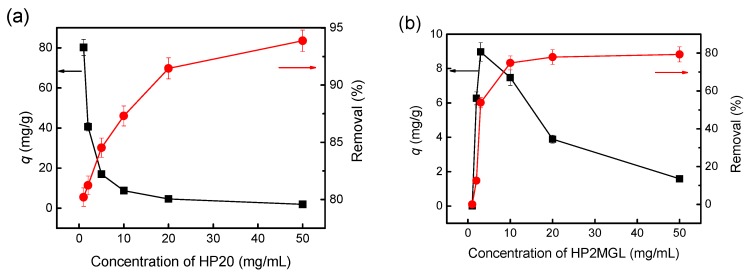
Effect of the concentrations of (**a**) HP20 and (**b**) HP2MGL on adsorption efficiency of SDBS. [SDBS] = 0.1 mg/mL; adsorption time 7 h.

The effect of the amount of adsorbents on the adsorbed amount (*q*) and removal of SBDS was investigated at an SDBS concentration of 0.1 mg/mL after 7 h adsorption time (Figure 4). The *q* value and the removal efficiency were much higher for HP20 than for HP2MGL (more than 10 times for *q*, and about 20% higher removal efficiency). A maximum amount of 80 mg/g SDBS was adsorbed with 1 mg/mL HP20. The efficiency of SDBS removal using HP20 increased from 80 to 94% with an increase in the amount of HP20 used (from 1 to 50 mg/mL), and a lower maximum efficiency of SDBS removal (80%) was observed when using HP2MGL under the same conditions. In addition, the maximum amount of SDBS adsorbed by HP2MGL was only 9 mg/g when 3 mg/mL of HP2MGL was used. The large difference in the adsorption indicated that HP20 had a better adsorption capability for SDBS than HP2MGL, presumably because of the interaction between SDBS and the phenyl rings in St in HP20, which the MMA-based HP2MGL does not contain. The same behavior was reported, describing that a molecule having aromatic structure could be adsorbed via strong electron π–π interaction between adsorbate and adsorbent [23].

### 3.4. Effect of Initial SDBS Concentration

**Figure 5:**
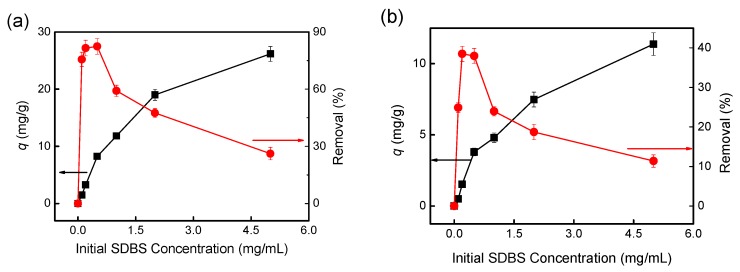
Effect of initial SDBS concentration on the removal of SDBS using (**a**) HP20 and (**b**) HP2MGL. [adsorbents] = 5 mg/mL; adsorption time = 7 h.

To investigate the effect of the initial SDBS concentration on *q* and the removal efficiency, various initial SDBS concentrations were used ranging from 0.5 to 5 mg/mL (Figure 5). The amount of SDBS adsorbed on MARs (*q*) increased with the initial SDBS concentration. The removal efficiency was maximized at a 0.5 mg/mL initial concentration of SDBS and then gradually decreased. Comparing both MARs, HP20 showed better removal ability (26 mg/g) at an initial SDBS concentration of 5 mg/mL, whereas SDBS removal by HP2MGL was less effective (11.3 mg/g). The results also suggest that the phenyl rings in HP20 were essential in providing a specific interaction with SDBS. The critical micelle concentration (CMC) of SDBS has been reported to range from 553 to 1400 μg/mL in the literature [24,25]. The *q* value was not affected by the CMC of SDBS, although the mechanism of adsorption of surfactants above the CMC has not been clarified [26].

### 3.5. Removal of SDBS from a Mixed Solution of SDBS and Cs Ions

**Figure 6:**
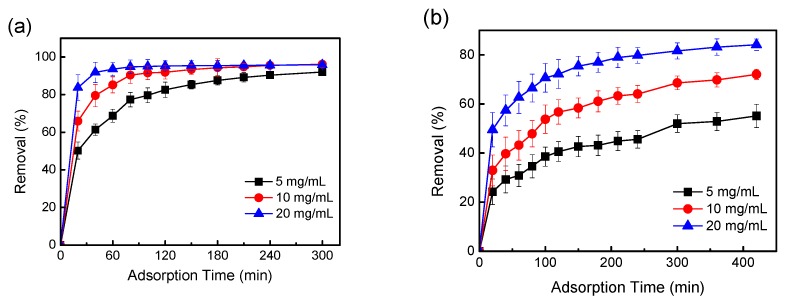
Changes in SDBS adsorption efficiency of (**a**) HP20 and (**b**) HP2MGL in SDBS/Cs with adsorption time at various concentrations of adsorbents. [SDBS] = 0.1 mg/mL.

Large amounts of surfactants are used to decontaminate RLWs, and thus large amounts of radioactive complexes are generated during decontamination and decommissioning processes. To simulate such radioactive complexes, we prepared a mixed solution consisting of SDBS and Cs ions (denoted as SDBS/Cs). The effect of the adsorption time on SDBS removal from the aqueous SDBS/Cs solution using various amounts of the MARs is shown in Figure 6. Interestingly, HP20 (20 mg/mL) showed a better SDBS removal efficiency from the SDBS/Cs solution than from the pure SDBS solution: more than 90% of the SDBS was removed in 40 min (Figure 6a). The removal of SDBS from the SDDBS/Cs solution by HP20 was noticeably more efficient than that by HP2MGL (Figure 6b). After 60 min adsorption time, the efficiency of SDBS removal reached equilibrium at 95% (by a 20 mg/mL HP20) after 30 min and at 80% (by a 20 mg/mL HP2MGL) after 240. SDBS, an anionic surfactant, can form a complex with metal (Cs ions) via an electrostatic interaction between the negatively charged anionic surfactants and the positively charged metal ions [27]. It is assumed that the complex became more hydrophobic than the anionic surfactant alone in aqueous solution, because of charge compensation. Therefore, the increase in the hydrophobicity of SDBS/Cs complexes resulted in better adsorption capacity for HP20 and less efficient adsorption for HP2MGL.

**Figure 7:**
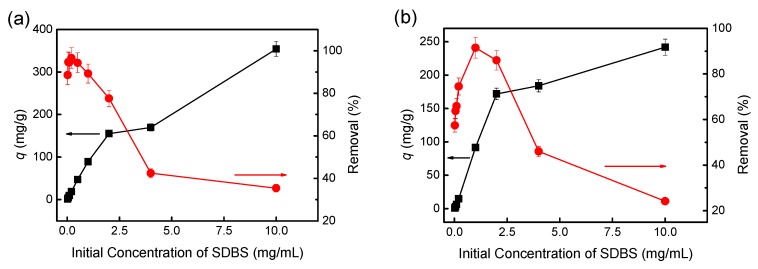
Effect of initial SDBS concentration for the removal of SDBS by (**a**) HP20 and (**b**) HP2MGL from SDBS/Cs. [adsorbents] = 5 mg/mL; adsorption time = 7 h.

In addition, the adsorption of various initial concentrations of SDBS from the SDBS/Cs solution by the MARs was significantly enhanced, compared with the pure SDBS solution (Figure 7). The maximum amount of SDBS adsorbed by the MARs increased more than five times (compared with *q* in Figure 5). When using HP20 for the removal of SDBS from SDBS/Cs solution, the maximum adsorption of SDBS observed was as 345 mg/g (for a 10 mg/mL initial SDBS concentration in SDBS/Cs aqueous solution). This is remarkably high removal of SDBS in the presence of metal ions in comparison with a previous report (140 mg/g from SDBS/Ca solution) [25]. Because of the difference in the hydrophobic nature of the resins, HP2MGL showed inefficient adsorption, compared with HP20.

**Figure 8:**
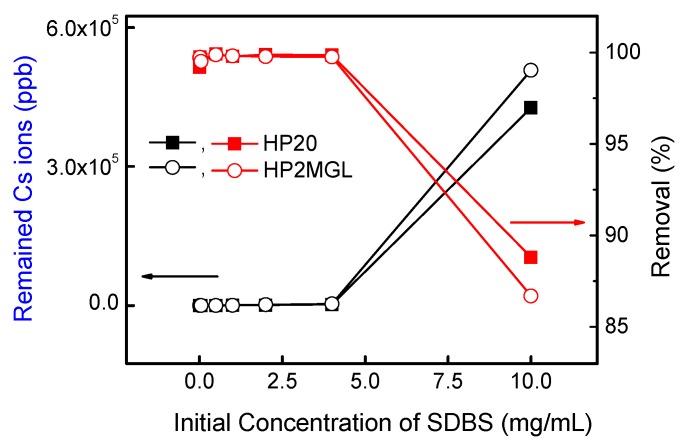
Removal efficiency of Cs ions with SDBS by HP20 and HP2MGL from SDBS/Cs at various initial concentrations of SDBS. [adsorbents] = 5 mg/mL; adsorption time 7 h.

To investigate whether Cs ions were removed by the resins from SDBS/Cs solution simultaneously with SDBS, the concentration of Cs ions that remained after adsorption by the MARs was measured. More than 85% of Cs ion removal was attained by both MARs (Figure 8). This result suggests that when the SDBS was removed from SDBS/Cs solution by the MARs, Cs ions were removed together with the SDBS.

### 3.6. Adsorption Isotherm

To elucidate the adsorption behavior of SDBS on MARs, Langmuir and Freundlich isotherm models were investigated [28,29]. Langmuir isotherm model is for a type of chemical process, in which the target molecules are adsorbed as a monolayer onto the surface of adsorbents. Langmuir isotherm equation is described below:

(2) $$\frac{C_{e}}{q_{e}}=\frac{1}{Kq_{\max}}+\frac{C_{e}}{q_{\max}}$$

where *C*_e_ (mg/mL) and *q*_e_ (mg/g) are the equilibrium concentration of SDBS and the adsorption capacity at equilibrium, respectively. *q*_max_ and *K* are the maximum adsorption capacity (mg/g) and the equilibrium adsorption constant (mL/mg), respectively, calculated by the fitting curve.

Freundlich isotherm model suggests that the multilayer-adsorption process of target molecules on the adsorbent surface. The Freundlich isotherm equation is represented by Equation (3):

(3) $$log\;q_{e}=log\;K_{F}+\frac{1}{n}log\;C_{e}$$

where *C*_e_ (mg/mL) and *q*_e_ (mg/g) are the equilibrium concentration of SDBS and the adsorption capacity at equilibrium. *K_F_* and 1/*n* are the adsorption constants of Freundlich isotherm model that mean the adsorption capacity (for *K_F_*) and the adsorption intensity (for 1/*n*).

**Figure 9:**
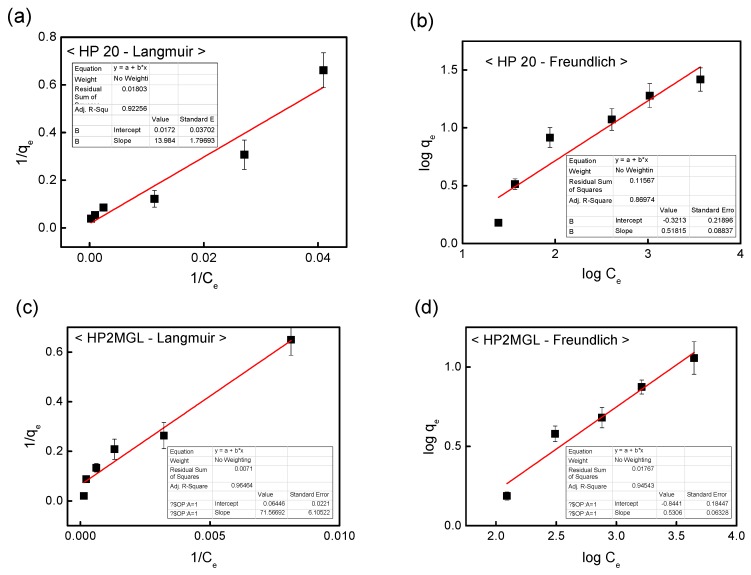
Langmuir (**a**,**c**) and Freundlich isotherm models (**b**,**d**) for the adsorption of SDBS onto MARs, HP20 (**a**,**b**) and HP2MGL (**c**,**d**). [adsorbents] = 5 mg/mL; [SDBS] = 0.1–5 mg/mL; adsorption time 7 h.

The adsorption behavior of SDBS on MARs and the adsorption capacities of MARs were explored using Langmuir and Freundlich isotherm models. Figure 9 shows the fitting curves for both isotherm models, and the obtained values are tabulated in Table 2. The correlation value (*R*^2^) shows that the Freundlich model is good for explanation of adsorption with HP20, whereas, in the case of HP2MGL, the *R*^2^ showed that Langmuir model is suitable. The adsorption capacity of SDBS was calculated to be 57.9 (for HP20) and 15.5 mg/g (for HP2MGL), indicating that HP20 had better adsorption capability for SDBS than HP2MGL. Moreover, such an adsorption capacity of HP20 for removal of SDBS is higher than that used with other materials, such as activated carbon (29.4 mg/g), alumina (19.8 mg/g), and zeolite (30.7 mg/g) [12,30,31,32].

### 3.7. Desorption

**Figure 10:**
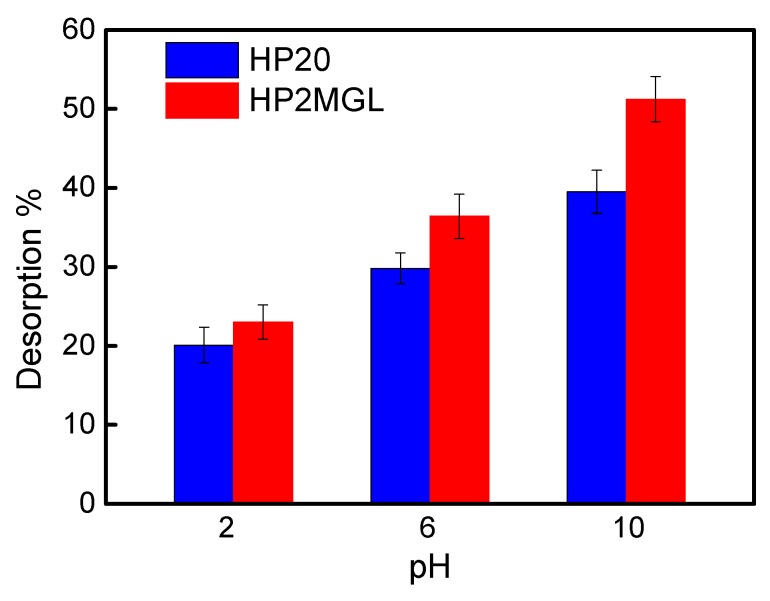
Desorption of SDBS from adsorbents in ultrapure water (10 mL) at various pHs (2, 6, and 10) at 25 °C. [adsorbents] = 5 mg/mL.

Desorption experiment should be carried out to elucidate the regeneration of adsorbent after use. Thus, the SDBS desorption efficiency of MARs was conducted at various pHs (2, 6, and 10) (Figure 10). HP20 with SDBS showed relatively lower desorption efficiency than that of HP2MGL at all pH ranges, and both MARs showed the gradual increase in the desorption efficiency with increase in the pH values. The SDBS could be removed from MARs by more than 40% after 2 h, providing that MARs could be regenerated via the desorption process for more than 2 h at high pH condition.

## 4. Conclusions

We demonstrated SDBS removal using two kinds of MARs, St- (for HP20) and MMA- (for HP2MGL)-based crosslinked polymers. The effects of adsorption time, the amount of adsorbents used, and the initial SDBS concentration on the SDBS adsorption using the resins were investigated in both solutions of pure SDBS and mixed solutions of SDBS and Cs ions. In the test of SDBS removal from the mixed solution of SDBS and Cs ions, which was an imitation of RLWs generated during decontamination, HP20 showed superb SDBS removal capability compared with HP2MGL. The maximum amount of SDBS adsorbed by HP20 (5 mg/mL) was 345 mg/g, observed at an initial SDBS concentration of 10 mg/m in the mixed SDBS/Cs solution. SDBS could be efficiently removed by the nonionic adsorbents, HP20 and HP2MGL used in this study, in which they were different from other materials reported previously in terms of surface hydrophilicity. The reported materials from previous studies adsorbed SDBS via hydrophilic interaction, ionic interaction, or chemical bond with oxygen [33,34]. However, the MARs in this study did not contain ionic functional groups and thus could remove SDBS via hydrophobic interaction and π–π interaction. The results proved that the MARs were able to remove SDBS not only from pure SDBS solution but also from SDBS/Cs solution, indicating their potential usefulness for treating RLWs generated from decontamination processes.

## Figures and Tables

**Table 1:** Surface Properties of HP20 and HP2MGL.

Adsorbents	Surface Area (m^2^/g)	Pore Volume (mL/g)	Pore Radius (Å)
HP20	614.1642	1.0140	66.0466
HP2MGL	594.0658	1.0862	73.1393

**Table 2:** Langmuir and Freundlich Parameters for the adsorption of SDBS Using HP20 and HP2MGL.

Model	Isotherm Constants	Values	
		HP20	HP2MGL
**Langmuir**	*q*_max_ (mg g^−1^)	57.9	15.5
	*K* (mL mg^−1^)	0.001	0.0009
	*R* ^2^	0.879	0.965
**Freundlich**	*K_F_* (mg g^−1^ (mL mg^−1^)^1/n^)	0.477	0.143
	*n*	1.930	1.885
	*R* ^2^	0.923	0.945
